# Crystal structure of (*E*)-1-(3-chloro­phen­yl)-3-(furan-2-yl)prop-2-en-1-one

**DOI:** 10.1107/S2056989015016266

**Published:** 2015-09-12

**Authors:** Sarah K. Zingales, Maya Z. Wallace, Clifford W. Padgett

**Affiliations:** a Armstrong State University, Department of Chemistry and Physics, 11935 Abercorn St., Savannah, GA 31419, USA

**Keywords:** crystal structure, chalcone

## Abstract

The title compound, C_13_H_9_ClO_2_, exhibits a non-planar geometry; the furan ring being inclined to the benzene ring by 50.52 (16)°. In the crystal, mol­ecules stack along the *a* axis; however, there are no significant inter­molecular inter­actions present.

## Related literature   

For the synthesis of the title compound, see: HanLee *et al.* (2011 ▸). For the syntheses of related compounds, see: Inokuma *et al.* (2009 ▸).
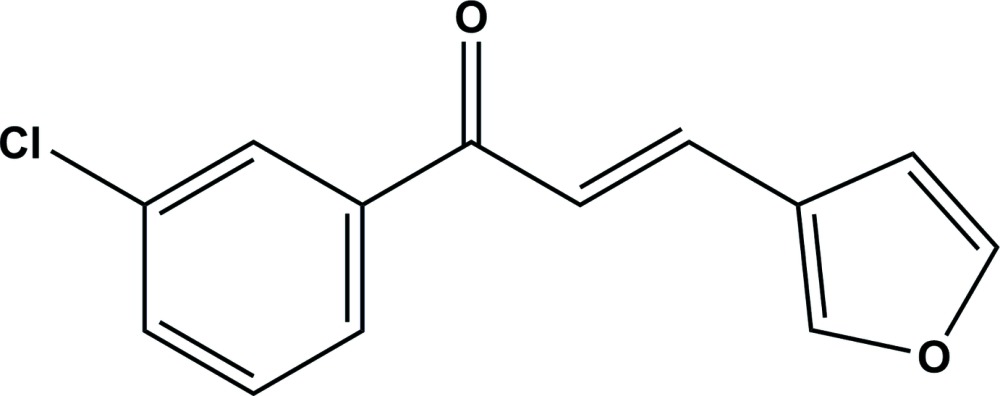


## Experimental   

### Crystal data  

C_13_H_9_ClO_2_
*M* *_r_* = 232.67
Monoclinic, 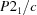

*a* = 7.186 (8) Å
*b* = 25.77 (3) Å
*c* = 5.774 (6) Å
β = 94.734 (10)°
*V* = 1066 (2) Å^3^
*Z* = 4
Mo *K*α radiation
μ = 0.34 mm^−1^
*T* = 173 K
0.30 × 0.30 × 0.10 mm

### Data collection  

Rigaku XtaLAB mini diffractometer
Absorption correction: multi-scan (REQAB; Rigaku, 1998 ▸) *T*_min_ = 0.832, *T*_max_ = 0.967
11328 measured reflections
2461 independent reflections
1672 reflections with *F* ^2^ > 2.0σ(*F* ^2^)
*R*_int_ = 0.080

### Refinement  

*R*[*F* ^2^ > 2σ(*F* ^2^)] = 0.054
*wR*(*F* ^2^) = 0.128
*S* = 1.07
2461 reflections
145 parameters
H-atom parameters constrained
Δρ_max_ = 0.29 e Å^−3^
Δρ_min_ = −0.33 e Å^−3^

### 

Data collection: *CrystalClear-SM Expert* (Rigaku, 2011 ▸); cell refinement: *CrystalClear-SM Expert*; data reduction: *CrystalClear-SM Expert*; program(s) used to solve structure: *SHELXS97* (Sheldrick, 2008 ▸); program(s) used to refine structure: *SHELXL97* (Sheldrick, 2008 ▸); molecular graphics: *CrystalStructure* (Rigaku, 2011 ▸); software used to prepare material for publication: *CrystalStructure*.

## Supplementary Material

Crystal structure: contains datablock(s) Global, I. DOI: 10.1107/S2056989015016266/su5196sup1.cif

Structure factors: contains datablock(s) I. DOI: 10.1107/S2056989015016266/su5196Isup2.hkl

Supporting information file. DOI: 10.1107/S2056989015016266/su5196Isup3.tif

Supporting information file. DOI: 10.1107/S2056989015016266/su5196Isup4.cml

**Fig. 1.**
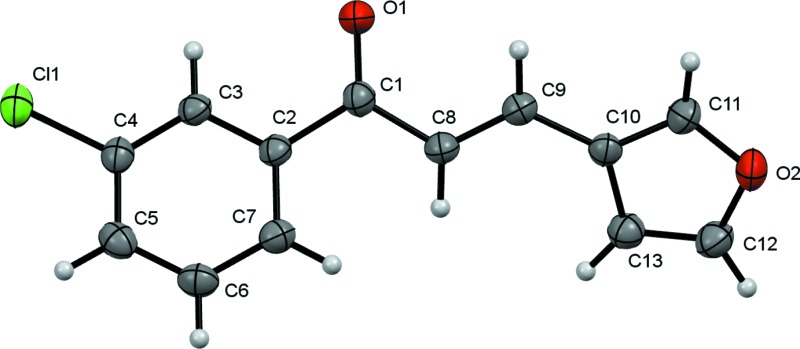
A view of the molecular structure of the title compound, with atom labelling. Displacement ellipsoids are drawn at the 50% probability level.

. DOI: 10.1107/S2056989015016266/su5196fig1.tif
A view of the mol­ecular structure of the title compound, with atom labelling. Displacement ellipsoids are drawn at the 50% probability level.

CCDC reference: 1421581

Additional supporting information:  crystallographic information; 3D view; checkCIF report

## supplementary crystallographic information

### S1. Chemical context

The title compound is a chalcone analog that is currently being studied for its potential anti­cancer activity [unpublished results]. Its synthesis and NMR characteristics have been reported (HanLee *et al.* 2011), but its crystal structure has not been reported until now.

### S2. Synthesis and crystallization

The title compound was synthesized by an aldol condensation reaction. 3-furaldehyde (1 mmol) and 3-chloro­aceto­phenone (1 mmol) were dissolved in ethanol (5 ml). A NaOH solution (5 M, 1 mL) was added and the reaction mixture was stirred until a precipitate formed. The reaction mixture was cooled in an ice bath for 20 min. The solids formed were filtered off and recrystallized from MeOH/H_2_O (1:1, v:v). Slow evaporation of a solution of the title compound in MeOH gave pale brown crystals.

### S3. Refinement

Crystal data, data collection and structure refinement details are summarized in Table 1. The H atoms were included in calculated positions and treated as riding atoms: C—H = 0.95 Å with U_iso_(H) = 1.2U_eq_(C).

### Crystal data

**Table:**

C_13_H_9_ClO_2_	*F*(000) = 480.00
*M**_r_* = 232.67	*D*_x_ = 1.450 Mg m^−^^3^
Monoclinic, *P*2_1_/*c*	Mo *K*α radiation, λ = 0.71075 Å
Hall symbol: -P 2ybc	Cell parameters from 2235 reflections
*a* = 7.186 (8) Å	θ = 2.4–27.5°
*b* = 25.77 (3) Å	µ = 0.34 mm^−^^1^
*c* = 5.774 (6) Å	*T* = 173 K
β = 94.734 (10)°	Prism, colorless
*V* = 1066 (2) Å^3^	0.30 × 0.30 × 0.10 mm
*Z* = 4	

### Data collection

**Table:**

Rigaku XtaLAB mini diffractometer	1672 reflections with *F*^2^ > 2.0σ(*F*^2^)
Detector resolution: 6.827 pixels mm^-1^	*R*_int_ = 0.080
ω scans	θ_max_ = 27.6°
Absorption correction: multi-scan (REQAB; Rigaku, 1998)	*h* = −9→9
*T*_min_ = 0.832, *T*_max_ = 0.967	*k* = −33→33
11328 measured reflections	*l* = −7→7
2461 independent reflections	

### Refinement

**Table:**

Refinement on *F*^2^	Secondary atom site location: difference Fourier map
*R*[*F*^2^ > 2σ(*F*^2^)] = 0.054	Hydrogen site location: inferred from neighbouring sites
*wR*(*F*^2^) = 0.128	H-atom parameters constrained
*S* = 1.07	*w* = 1/[σ^2^(*F*_o_^2^) + (0.0293*P*)^2^ + 0.6811*P*] where *P* = (*F*_o_^2^ + 2*F*_c_^2^)/3
2461 reflections	(Δ/σ)_max_ < 0.001
145 parameters	Δρ_max_ = 0.29 e Å^−^^3^
0 restraints	Δρ_min_ = −0.33 e Å^−^^3^
Primary atom site location: structure-invariant direct methods	

### Special details

**Table:**

Refinement. Refinement was performed using all reflections. The weighted *R*-factor (*wR*) and goodness of fit (*S*) are based on *F*^2^. *R*-factor (gt) are based on *F*. The threshold expression of *F*^2^ > 2.0 σ(*F*^2^) is used only for calculating *R*-factor (gt).

### Fractional atomic coordinates and isotropic or equivalent isotropic displacement parameters (Å^2^)

**Table:**

	*x*	*y*	*z*	*U*_iso_*/*U*_eq_	
Cl1	0.25413 (11)	0.29816 (3)	0.35320 (13)	0.0404 (3)	
O1	0.2759 (3)	0.50459 (7)	0.3133 (4)	0.0385 (6)	
O2	0.3111 (3)	0.71261 (7)	0.9248 (4)	0.0395 (5)	
C1	0.2650 (4)	0.49620 (10)	0.5220 (5)	0.0295 (6)	
C2	0.2198 (4)	0.44238 (10)	0.6022 (5)	0.0263 (6)	
C3	0.2537 (4)	0.40038 (10)	0.4594 (5)	0.0271 (6)	
C4	0.2074 (4)	0.35117 (10)	0.5274 (5)	0.0289 (6)	
C5	0.1236 (4)	0.34244 (11)	0.7323 (5)	0.0335 (7)	
C6	0.0897 (4)	0.38439 (11)	0.8733 (5)	0.0334 (7)	
C7	0.1396 (4)	0.43435 (11)	0.8108 (5)	0.0304 (7)	
C8	0.2914 (4)	0.53727 (10)	0.6976 (5)	0.0293 (6)	
C9	0.2727 (4)	0.58734 (10)	0.6361 (5)	0.0271 (6)	
C10	0.3055 (4)	0.63097 (10)	0.7933 (5)	0.0275 (6)	
C11	0.2624 (4)	0.68115 (11)	0.7414 (5)	0.0338 (7)	
C12	0.3882 (5)	0.68125 (12)	1.0974 (5)	0.0369 (7)	
C13	0.3873 (4)	0.63144 (11)	1.0275 (5)	0.0327 (7)	
H3	0.3082	0.4057	0.3168	0.0325*	
H5	0.0902	0.3083	0.7751	0.0402*	
H6	0.0320	0.3790	1.0135	0.0400*	
H7	0.1190	0.4628	0.9100	0.0365*	
H8	0.3220	0.5284	0.8559	0.0352*	
H9	0.2353	0.5948	0.4780	0.0325*	
H11	0.2058	0.6927	0.5960	0.0406*	
H12	0.4359	0.6930	1.2466	0.0443*	
H13	0.4323	0.6023	1.1162	0.0393*	

### Atomic displacement parameters (Å^2^)

**Table:**

	*U*^11^	*U*^22^	*U*^33^	*U*^12^	*U*^13^	*U*^23^
Cl1	0.0503 (5)	0.0258 (4)	0.0454 (5)	0.0004 (4)	0.0067 (4)	−0.0059 (3)
O1	0.0601 (15)	0.0292 (11)	0.0266 (11)	0.0010 (10)	0.0055 (10)	0.0005 (9)
O2	0.0520 (13)	0.0243 (11)	0.0418 (12)	−0.0012 (10)	0.0011 (10)	−0.0057 (9)
C1	0.0321 (15)	0.0254 (14)	0.0308 (15)	0.0027 (12)	0.0017 (12)	−0.0006 (12)
C2	0.0285 (14)	0.0268 (14)	0.0231 (13)	0.0008 (12)	−0.0017 (11)	−0.0017 (11)
C3	0.0289 (15)	0.0297 (15)	0.0224 (14)	−0.0024 (12)	0.0002 (11)	−0.0026 (11)
C4	0.0294 (15)	0.0258 (14)	0.0308 (15)	0.0019 (12)	−0.0023 (12)	−0.0024 (12)
C5	0.0319 (16)	0.0331 (16)	0.0347 (16)	−0.0032 (13)	−0.0026 (13)	0.0051 (12)
C6	0.0314 (16)	0.0391 (17)	0.0302 (15)	−0.0022 (13)	0.0065 (12)	0.0035 (13)
C7	0.0311 (15)	0.0339 (16)	0.0262 (14)	0.0024 (13)	0.0023 (12)	−0.0014 (12)
C8	0.0351 (16)	0.0285 (14)	0.0239 (14)	0.0001 (13)	−0.0001 (11)	0.0000 (11)
C9	0.0272 (14)	0.0294 (14)	0.0247 (14)	0.0002 (12)	0.0022 (11)	−0.0014 (11)
C10	0.0287 (14)	0.0271 (14)	0.0275 (14)	−0.0018 (12)	0.0062 (11)	−0.0008 (11)
C11	0.0397 (17)	0.0307 (15)	0.0304 (15)	−0.0047 (13)	−0.0009 (13)	−0.0035 (12)
C12	0.0419 (18)	0.0388 (17)	0.0295 (15)	0.0016 (15)	−0.0007 (13)	−0.0049 (13)
C13	0.0338 (16)	0.0335 (16)	0.0307 (15)	0.0020 (14)	0.0008 (12)	0.0002 (12)

### Geometric parameters (Å, º)

**Table:**

Cl1—C4	1.746 (3)	C4—C5	1.389 (5)
O1—C1	1.233 (4)	C5—C6	1.387 (5)
O2—C11	1.357 (4)	C6—C7	1.392 (5)
O2—C12	1.365 (4)	C8—C9	1.342 (4)
C1—C2	1.506 (4)	C9—C10	1.452 (4)
C1—C8	1.467 (4)	C10—C11	1.357 (4)
C2—C3	1.394 (4)	C10—C13	1.429 (4)
C2—C7	1.393 (4)	C12—C13	1.346 (5)
C3—C4	1.377 (4)		
			
C11—O2—C12	106.2 (3)	C4—C5—C6	118.9 (3)
O1—C1—C2	119.7 (3)	C5—C6—C7	120.5 (3)
O1—C1—C8	122.3 (3)	C2—C7—C6	119.7 (3)
C2—C1—C8	118.1 (3)	C1—C8—C9	120.4 (3)
C1—C2—C3	118.7 (3)	C8—C9—C10	124.9 (3)
C1—C2—C7	121.2 (3)	C9—C10—C11	125.3 (3)
C3—C2—C7	120.1 (3)	C9—C10—C13	129.1 (3)
C2—C3—C4	119.2 (3)	C11—C10—C13	105.6 (3)
Cl1—C4—C3	119.6 (3)	O2—C11—C10	110.9 (3)
Cl1—C4—C5	118.8 (3)	O2—C12—C13	110.7 (3)
C3—C4—C5	121.6 (3)	C10—C13—C12	106.5 (3)
