# Supplementary figures and images for: Crystal structure of (E)-1-(3-chloro­phen­yl)-3-(furan-2-yl)prop-2-en-1-one
Source: Acta Crystallogr E Crystallogr Commun. 2015 Sep 12;71(Pt 10):o707. doi: 10.1107/S2056989015016266 (PMC4647362; doi:10.1107/S2056989015016266)

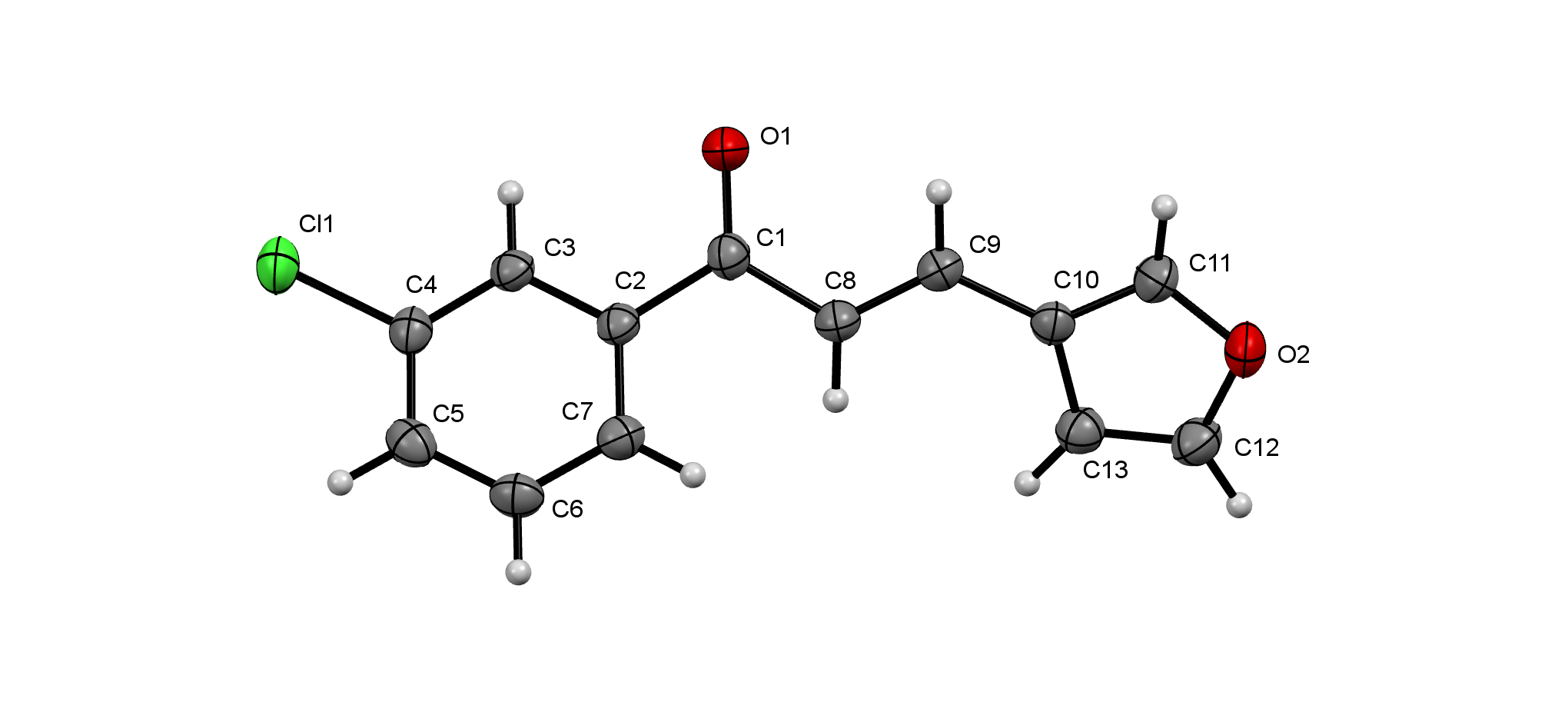

Supplement: Supplementary file 3 [file e-71-0o707-Isup3.tif]

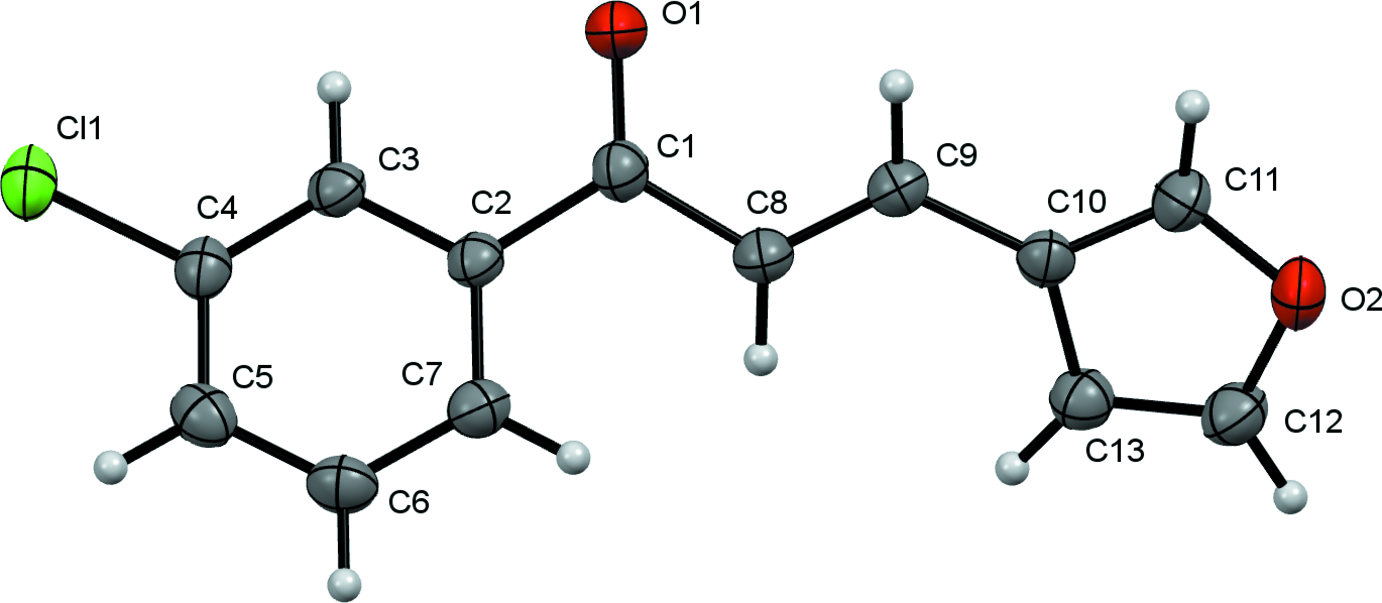

Supplement: Supplementary file 5 [file e-71-0o707-fig1.tif]
